# Diversity of the Photoreceptors and Spectral Opponency in the Compound Eye of the Golden Birdwing, *Troides aeacus formosanus*

**DOI:** 10.1371/journal.pone.0062240

**Published:** 2013-04-16

**Authors:** Pei-Ju Chen, Kentaro Arikawa, En-Cheng Yang

**Affiliations:** 1 Department of Entomology, National Taiwan University, Taipei, Taiwan; 2 Laboratory of Neuroethology, Sokendai-Hayama (The Graduate University for Advanced Studies), Hayama, Japan; 3 Graduate Institute of Brain and Mind Sciences, National Taiwan University, Taipei, Taiwan; Monash University, Australia

## Abstract

The compound eye of the Golden Birdwing, *Troides aeacus formosanus* (Papilionidae, Lepidoptera), is furnished with three types of ommatidia, which are clearly different in pigmentation around the rhabdom. Each ommatidium contains nine photoreceptors, whose spectral sensitivities were analyzed electrophysiologically. We identified nine spectral types of photoreceptor with sensitivities peaking at 360 nm (UV), 390 nm (V), 440 nm (B), 510 nm (BG), 540 nm (sG), 550 nm (dG), 580 nm (O), 610 nm (R), and 630 nm (dR) respectively. The spectral sensitivities of the V, O, R and dR receptors did not match the predicted spectra of any visual pigments, but with the filtering effects of the pigments around the rhabdom, they can be reasonably explained. In some of the receptors, negative-going responses were observed when they were stimulated at certain wavelengths, indicating antagonistic interactions between photoreceptors.

## Introduction

Color vision is defined as the capability to discriminate visual stimuli based solely on the difference in spectral distribution, independent of the stimulus intensity [1]. In terms of physiology, the existence of at least two spectral types of photoreceptor in the retina is a prerequisite for color vision [2]. To achieve better discrimination, several strategies have been developed to enhance the spectral resolution in the visual system, such as narrowing the spectral sensitivity, shifting the peak sensitivity, and diversifying the spectral type of photoreceptor [3].

The absorption spectrum of visual pigments is the primary factor in determining the spectral sensitivity of photoreceptors, but it is largely modified by some other factors. The spectral sensitivity can be widened by the self screening effect of the visual pigment in the same photoreceptor [4], or by coexpressing multiple visual pigments in a photoreceptor [5], [6]. It can also be sharpened by the lateral filtering of neighboring photoreceptors [7], or by the filtering effect of fluorescing and colored pigments [8], [9]. In addition to the optical filtering by visual and non-visual pigments, the electrical interaction between photoreceptors can also modify the spectral sensitivity [10]. Electrical interactions between photoreceptors have been reported in the retina of the Australian orchard butterfly, *Papilio aegeus* [11], [12], where some photoreceptors depolarize at certain wavelengths and hyperpolarize at different wavelengths. The hyperpolarization, or negative-going responses, may originate from other simultaneously stimulated photoreceptors, causing an inhibitory effect on the photoreceptor in question [12].

As suggested by the number of literatures cited above, butterflies have been an important group of insects in the study of color vision. The first and the most extensively studied species is the Japanese yellow swallowtail, *Papilio xuthus* (tribe Papilionini, Papilionidae), whose eyes are furnished with at least six classes of spectral receptors [8]. Accumulated evidence has suggested that the spectral organization of butterfly eyes is quite diverse [13]–[16]. To address the question how such variability has evolved in butterflies, we have conducted a comparative study in representative species.

*Troides* is a genus of a peculiar group in Papilionidae, the birdwing butterflies, which are large with the wing span of more than 15 cm. The genus *Troides* contains 18 species. Although they all look similar with black forewings and bright yellow hindwings in both sexes, they still appear depending on vision when discriminating conspecific mates. We therefore have initiated a work on their vision in the Golden Birdwing, *Troides aeacus formosanus*. In the course of studying the spectral sensitivity of photoreceptors in the compound eye retina, we found nine spectrally distinct photoreceptors, which is the most for butterflies studied in this respect. We also frequently encountered photoreceptors that exhibit spectral opponency. In the present study we describe the various photoreceptors in *Troides aeacus formosanus*, with a particular focus on the optical filtering effects of various pigments as well as the physiological background of the negative-going responses in the spectrally-opponent photoreceptors.

## Materials and Methods

### Animals

Both sexes of the Golden Birdwing, *Troides aeacus formosanus*, were obtained from laboratory culture stock derived from eggs laid by females caught in the field around Taitung County, Taiwan. The hatched larvae were fed fresh *Aristolochia kankaoensis* leaves, and raised under a constant photo period (L∶ D = 8 h∶ 16 h) and temperature (25°C) in the laboratory. The butterflies were fed a sucrose solution daily and were used for experiments within two weeks after emergence.

### Electrophysiology

Photoreceptor spectral sensitivities were determined by intracellular electrophysiology [17].The wings and legs of the butterfly were amputated, and the thorax and the head were mounted on a metal pedestal with a beeswax and resin (3∶1) mixture. A silver wire was inserted into the stump of one of the antennae to serve as the reference electrode. To insert a glass micropipette into the eye, a small triangular hole covering about 10–20 facets was made in the dorsal region of eye and sealed with a drop of Vaseline in order to prevent coagulation of haemolymph. The eye was positioned at the center of a Cardan arm perimeter device set in a Faraday cage. The electrophysiological recording was performed in the dark so as to keep the butterfly nearly dark-adapted.

A glass micropipette filled with 2M potassium acetate, with resistance about 100–120 MΩ, was inserted vertically into the retina using a micromanipulator. After impaling a single photoreceptor, the ommatidium was stimulated with a series of monochromatic lights between 300–700 nm, with 10 nm steps, provided by a 1000 W xenon arc lamp through a monochromator (SP-150-M with 150-030-300 grating, Acton Research Co.). The monochromatic light was guided through a quartz optical fiber providing a point light source of less than 0.4° in diameter. The terminal of the fiber was mounted on a Cardan arm perimeter device, accurately positioned at the point of maximum sensitivity of the cell being recorded. The intensity of the light stimulation was modulated over a range of 3 log units by a neutral density wedge (ND wedge). The duration of the flash stimulation for the spectral sensitivity measurement was limited to 50 ms by a shutter, and the interval between flashes was 15 s. A program to control the setup and record the responses was developed using LabVIEW software (ver. 8.2, National Instrument). The electrophysiological signals were amplified 10× by an amplifier and were acquired in real-time by a data-acquisition (DAQ) system (CA 1000 configurable signal conditioning enclosure, National Instruments).

First, a rough spectral scan (300–700 nm, 21 wavelengths with 20 nm steps) was performed with dim equiquantal flashes to identify the possible peak wavelength of the penetrated cell. A flash ramp test was then performed at the wavelength from low to high intensity and stopped when the response amplitude reached around 30–50% of the saturated response to “white light” stimulus. The intensity was adopted during the next measurement. A spectral scan (300–700 nm, 41 wavelengths with 10 nm steps) with equiquantal flashes was performed to measure the spectral responses and represented the means of two complete runs throughout the spectrum performed in opposite directions (from 300 to 700 nm and from 700 to 300 nm) to identify the peak wavelength (*λ*_max_). Then the response-light intensity (*V*-log *I*) function at the *λ*_max_ was recorded. The records were used only if the maximal response was over 30 mV, and were abandoned if the baseline potential shifted by more than 5 mV during the recording.

If the recorded photoreceptors showed a negative-going (hyperpolarizing) response in any wavelength range, the normalized angular responses and temporal impulse responses were recorded at the positive-peak and negative-peak wavelengths. For recording the angular responses, the Cardan arm was moved at 0.6 deg intervals. The response amplitude induced by a 50 ms stimulus at each angle of the field is referred to as the angular response. For recording the temporal impulse response, the shutter was set to deliver 20 ms light pulses at 5 s intervals. In order to compare the temporal properties of the positive and the negative responses, the ND wedge was adjusted to give a similar amplitude response around 2–3 mV of positive and negative responses using two wavelengths each eliciting either a positive or a negative response. The responses to 10 pulses were averaged for each cell. Finally, the time from the stimulus onset to the maximal response amplitude, *τ*_p_, of the positive and negative responses was compared.

The recorded *V*-log *I* data were fitted to the Naka-Rushton function [18]: *V*/*V*_max_ = *I* ^n^/(*I* ^n^ – *K* ^n^), where *I* is the stimulus intensity, *V* is the amplitude of responses, *V*_max_ is the peak amplitude at the maximum positive response, *K* is the stimulus intensity eliciting 50% *V*_max_, and n is the exponential slope. Amplitudes of the spectral responses were extrapolated to the *V*-log *I* function of a given unit to transform the amplitudes into photon numbers required for the responses. The normalized reciprocal of the relative photon numbers then yielded the spectral sensitivity.

### Anatomy

We first examined the intact eyes under the epi-fluorescence microscope (BX-60, Olympus) under UV excitation (dichroic cube U-MWU, excitation band-pass filter at 350 nm and emission cutoff filter at 420 nm). This was to check whether the *Troides* eyes contained fluorescing ommatidia like in other Papilionid species [15], [19]. For the histological investigation of the retina, the compound eyes were isolated and fixed in 8% paraformaldehyde in 2% sodium cacodylate buffer (pH 7.4) at room temperature for 30 min, dehydrated in an acetone series and embedded in Epon resin. The Epon-embedded samples were then sectioned into 10 µm thickness with a rotary microtome. The serial sections from the corneal surface to the bottom of the ommatidia were identified by normal transmission of white light to reveal their heterogeneity based on the pigmentation around the rhabdom.

### Cell marking

Some recorded photoreceptors were marked by the fluorescent dye Alexafluor 568 (excitation/emission at 576/599 nm) dissolved in 1M KCl (resistance about 120–150 MΩ). The dye was injected into the photoreceptor by applying a negative DC current of 2–5 nA for 5–10 min. Then the ommatidium containing the Alexafluor-injected receptor was identified with an epi-fluorescence microscope (BX-60, Olympus) under a 550 nm excitation (dichroic cube U-MWIG, excitation band-pass filter at 550 nm and emission cutoff filter at 570 nm). The eyes containing Alexafluor-injected photoreceptors were processed for histology as above, and the injected photoreceptor was localized within the ommatidial array.

## Results

### Ommatidial heterogeneity

**Figure 1:**
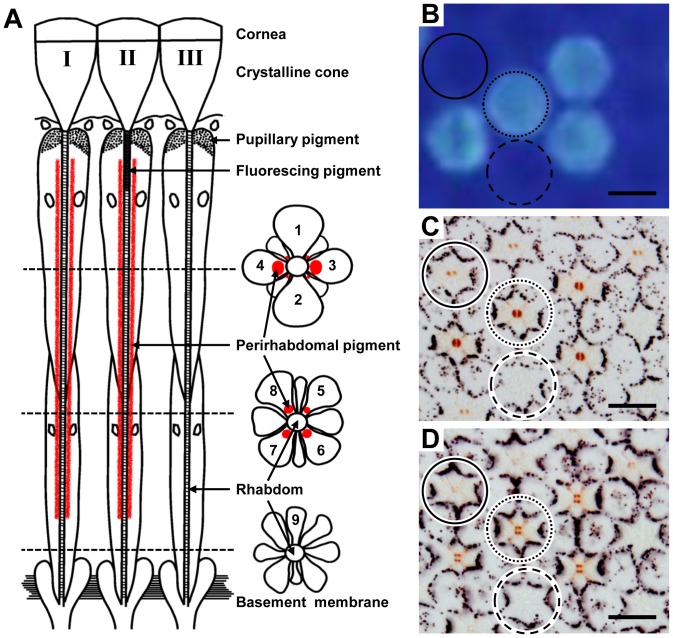
Ommatidial heterogeneity of *Troides aeacus formosanus*. (A) Scheme of three types of ommatidia: longitudinal (*left*) and transverse views at different levels (*right*). (B) Fluorescence micrograph of intact eye showing four ommatidia emit strong fluorescence (*dotted circle*). (C) Light micrograph of a transverse section at 200 µm from the corneal surface of the eye region shown in (B). The pigmentation around the rhabdom makes three ommatidial types distinguishable: type I (*solid circle*), II (*dotted circle*), and III (*dashed circle*). (D) The same specimen sectioned at 350 µm from the corneal surface shows the pigmentation in the proximal tier. *Scale bar* 20 µm.

The light microscope serial sections of the retina of *Troides aeacus formosanus* revealed that its compound eye is of a typical butterfly type, containing nine photoreceptor cells R1-9. Their rhabdomeres jointly constitute the fused rhabdom, with a possible segregation of the distal and proximal parts in the so-called tiered type rhabdom, where R1–4 are distal photoreceptors, R5–8 are proximal photoreceptors and R9 is the basal photoreceptor (Figure 1A).

In the transverse sections through the proximal tier, about 75% ommatidia have four pigment clusters, which are in the cell body of the R5–8 photoreceptors close to the rhabdom (Figure 1D). The arrangement of the four pigment clusters in R5–8 is trapezoidal (type I, 50% of the ommatidia) or square (type II, 25%). The pigmentation in other 25% of the ommatidia, type III, is not clear and may be absent. Types I and II ommatidia have red pigmentation, and also in R3 and R4 in the distal tier, which is however denser in type II than in type I (Figure 1C). The distribution of the three types of ommatidia is locally random. Fluorescence microscopy of an intact eye under UV epi-illumination revealed that 25% of the ommatidia are the fluorescing type. By combining fluorescence microscopy and histology, we found that the fluorescing ommatidia are type II (Figure 1B-D). Therefore, the three types of ommatidia are distinguishable by the characteristic pattern of fluorescence and red pigments.

### Spectral sensitivity of photoreceptors

**Figure 2:**
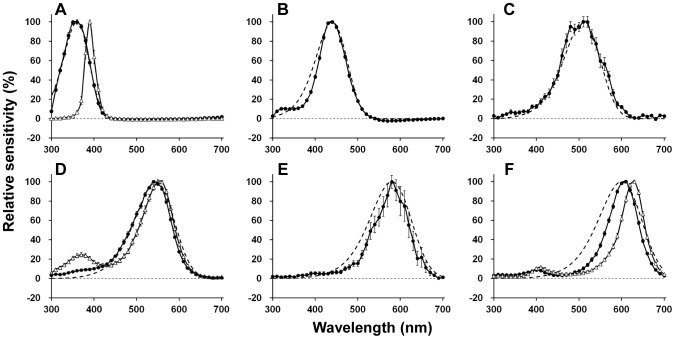
Spectral sensitivities of nine spectral receptor types found in the retina of *Troides aeacus formosanus*. The averaged spectral sensitivity curves are shown as *solid lines* with standard errors. The *dashed lines* indicate the predicted absorption spectra of visual pigment calculated from a template [22]. (A) UV (360 nm, n = 50) and violet (390 nm, n = 15) receptors with the absorption spectrum of a 360 nm-absorbing visual pigment (R360). (B) Blue (440 nm, n = 76) receptor with R440. (C) Blue-green (510 nm, n = 12) receptor with R510. (D) Single-peaked green (540 nm, n = 54) and dual-peaked green (550 nm, n = 31) receptors with R540. (E) Orange (580 nm, n = 4) receptor with R580. (F) Red (610 nm, n = 30) and deep-red (630 nm, n = 34) receptors with R600.

We obtained 306 successful recordings from dark adapted *Troides* retina. According to their peak wavelength (*λ*_max_), the photoreceptors are divided into nine types (Figure 2), peaking at 360 nm (ultraviolet (UV), n = 50), 390 nm (violet (V), n = 15), 440 nm (blue (B), n = 76), 510 nm (blue-green (BG), n = 12), 540 nm (single-peaked green (sG), n = 54), 550 nm (dual-peaked green (dG), n = 31), 580 nm (orange (O), n = 4), 610 nm (red (R), n = 30) and 630 nm (deep red (dR), n = 34). We could not detect any sexual difference except for the fact that we did not encounter the O receptor in females. The O receptor was encountered only infrequently in males.

Interpretation of sensitivities of UV and B receptors is simple because their spectral sensitivities are similar to the predicted absorption spectra of visual pigments peaking at 360 (R360) and 440 nm (R440), respectively (Figure 2A, B). Two types of G receptors, sG receptor peaking at 540 nm, and the dG receptor with the primary peak at 550 nm and a secondary peak at 370 nm (Figure 2D), are also rather simple. Regardless of the secondary peak, spectral sensitivity curves of sG and dG receptors both match with the absorption spectrum R540.

**Figure 3:**
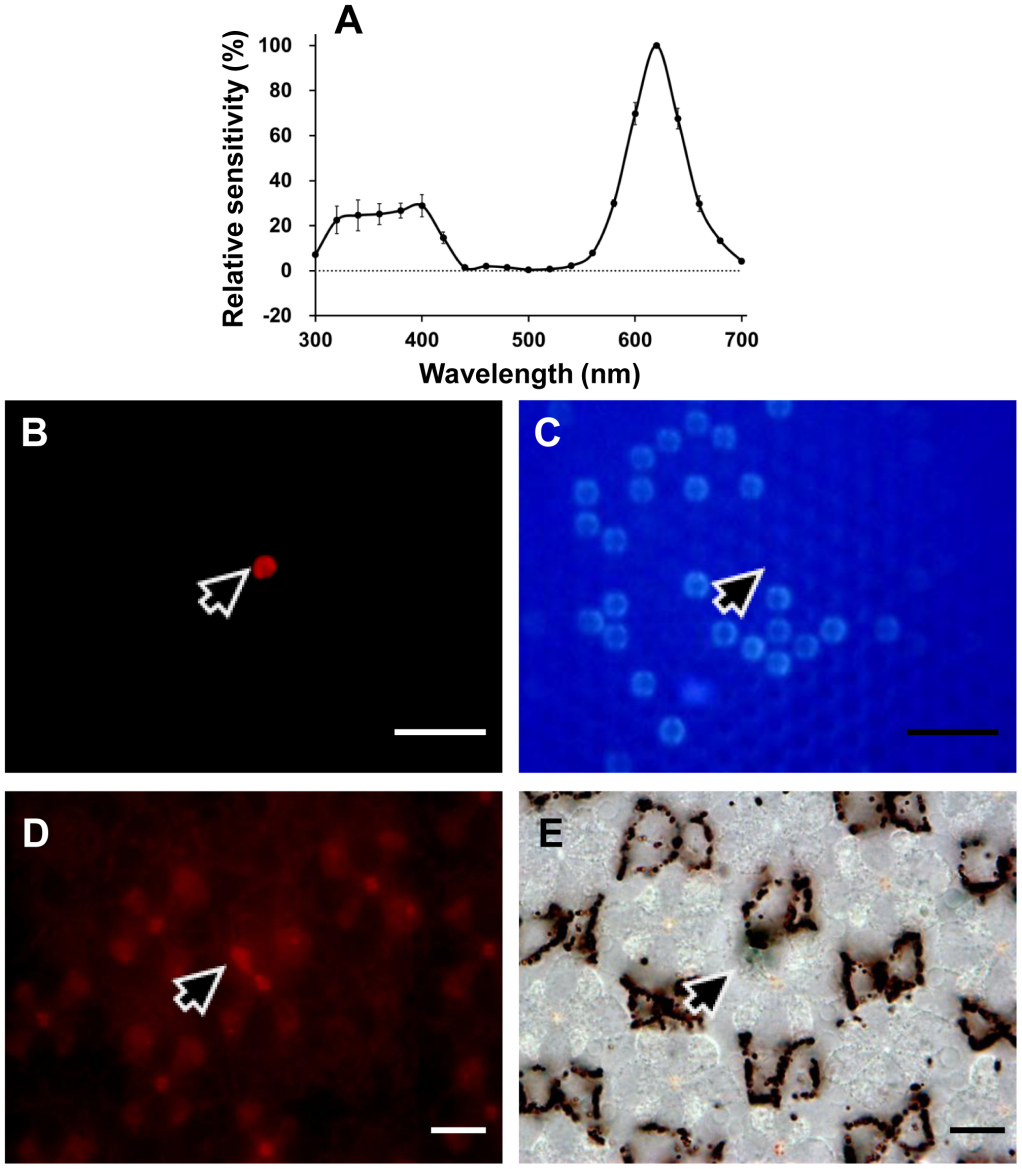
Localization of an R (610 nm) receptor. (A) The spectral sensitivity of the labeled photoreceptor (average of two spectral runs±standard errors). (B) Green-induced red fluorescence reveals the ommatidium (*arrow*) containing the Alexafluor-injected photoreceptor. (C) UV-induced fluorescence shows that the ommatidium (*arrow*) is type I. (D) A transverse section of the eye observed under green excitation shows that the photoreceptor labeled (*arrow*) is a proximal R8. (E) The same section under transmitted white light reveals that the ommatidium (*arrow*) is pale-red pigmented. *Scale bar* 100 µm in (B) and (C), 10 µm in (D) and (E).

**Figure 4:**
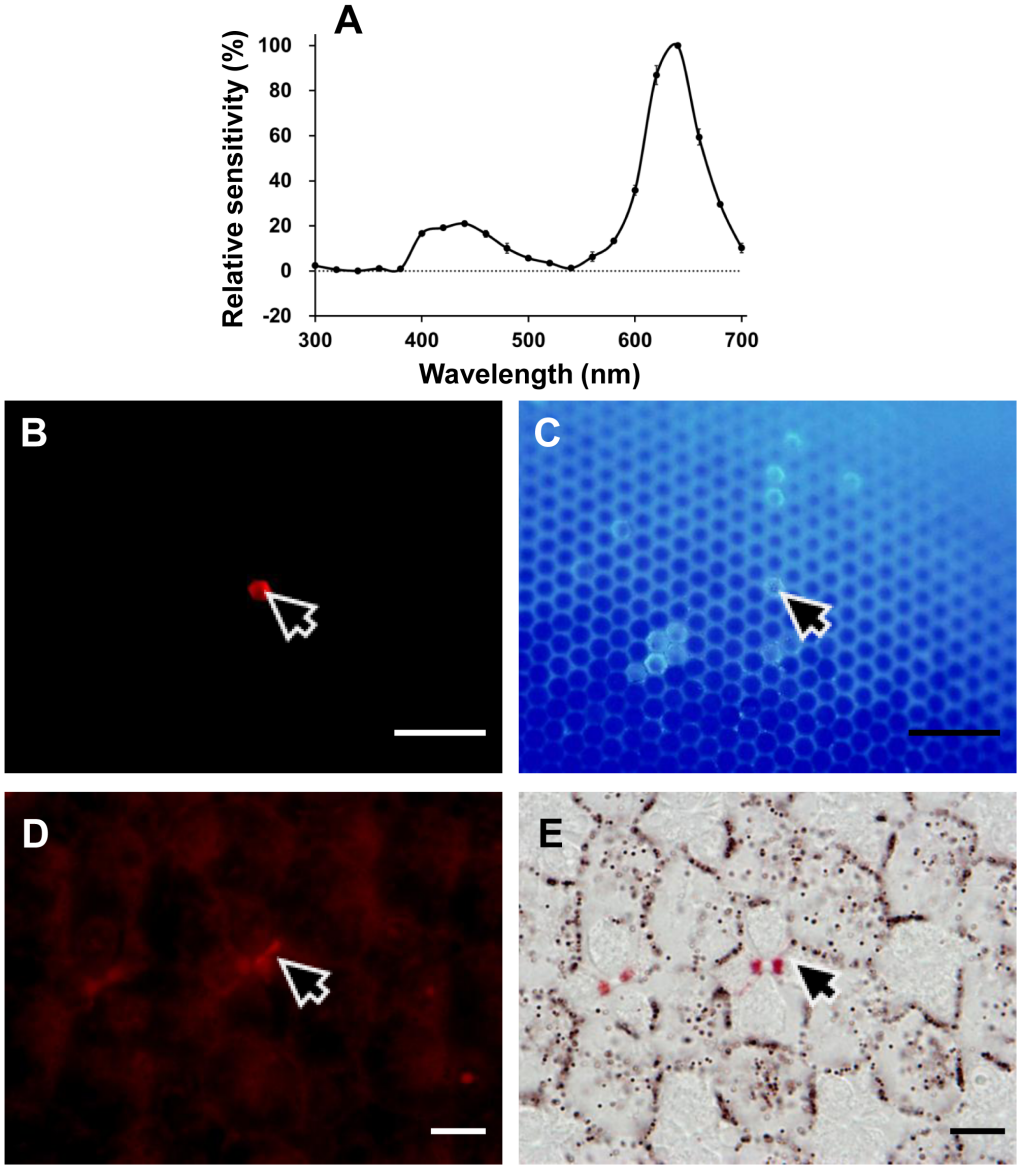
Localization of a dR (630 nm) receptor. (A) The spectral sensitivity of the labeled photoreceptor (average of two spectral runs±standard errors). (B) Green-induced red fluorescence reveals the ommatidium (*arrow*) containing the Alexafluor-injected photoreceptor. (C) UV-induced fluorescence shows that the ommatidium (*arrow*) is type II. (D) A transverse section of the eye observed under green excitation showing that the photoreceptor labeled (*arrow*) is a proximal R5. (E) The same section under transmitted white light reveals that the ommatidium (*arrow*) is deep-red pigmented. *Scale bar* 100 µm in (B) and (C), 10 µm in (D) and (E).

The spectral sensitivity of V receptors peaks at 390 nm (Figure 2A), and is much narrower than the predicted spectrum of any visual pigment. Such sharpness was also observed in the R and the dR receptor (Figure 2F). Both the R and dR receptors were successfully labeled and localized in the present study. The Alexafluor-injected R receptor was R8 in a type I ommatidium, which is non-fluorescing and red pigmented (Figure 3). Similarly, the Alexafluor-injected dR receptor was localized as R6 in a type II fluorescing ommatidium (Figure 4).

Two additional uncommon receptors were the BG receptors peaking at 510 nm (Figure 2C) and the O receptors peaking at 580 nm (Figure 2E). Their spectral sensitivity curves did not have a significant secondary peak in the UV range. The BG receptor has been recorded previously in both sexes, but to-date the O receptor has only been recorded in males probably because they are rare at any rate.

### Spatial and temporal properties of negative-going responses

**Figure 5:**
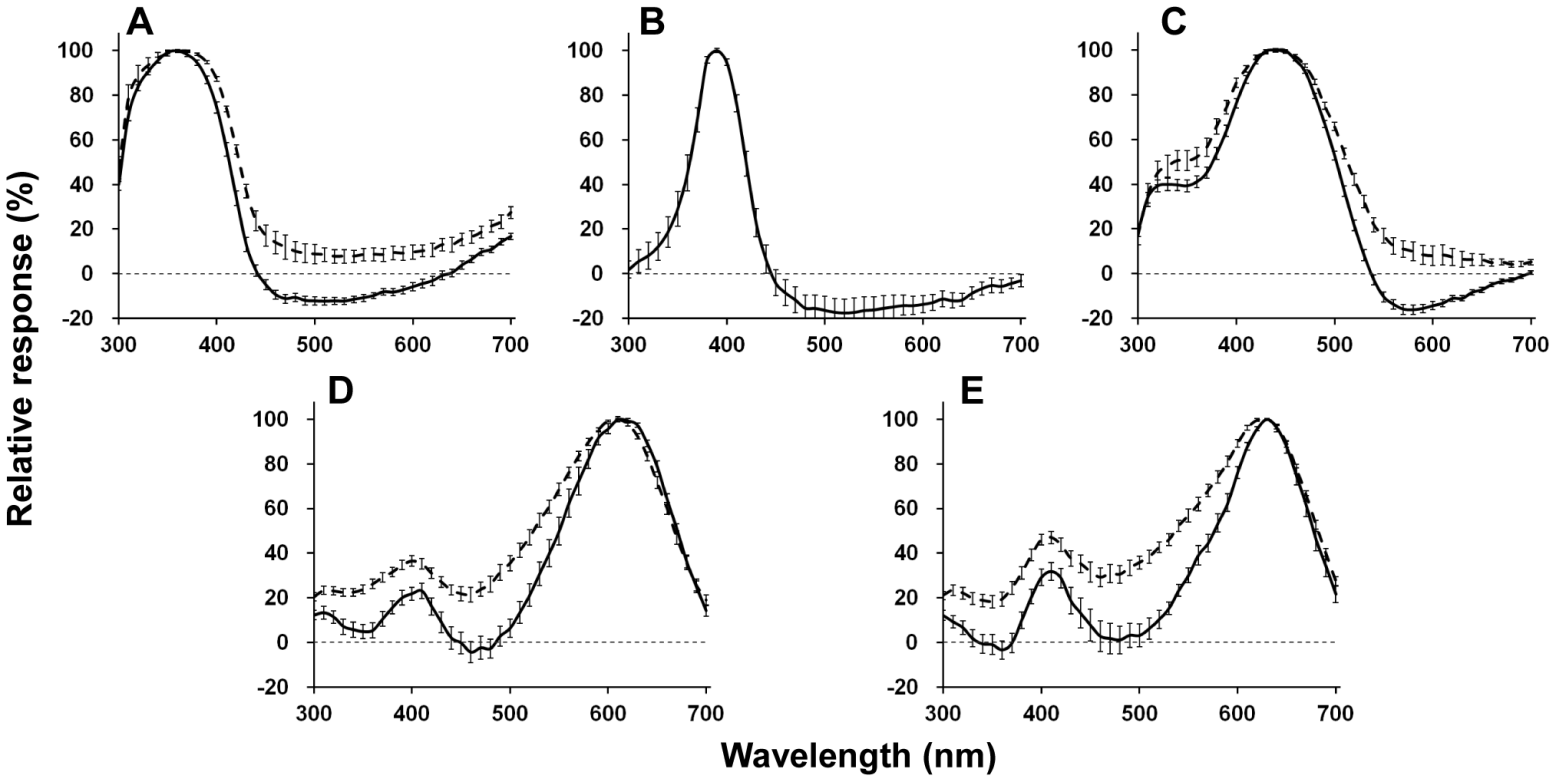
Averaged spectral response curves of photoreceptors with (*dotted lines*) and without (*solid lines*) negative-going responses. (A) UV receptor (number of cells with negative responses = 41/without negative responses = 9). (B) V receptor (15/0). (C) B receptor (67/9). (D) R receptor (11/19). (E) dR receptor (15/19). *Bars* indicate standard errors.

Among five spectral classes of photoreceptor (UV, V, B, R, and dR), quite a few cells showed negative-going responses when stimulated with specific wavelengths. The resting potentials of photoreceptors with negative-going responses to light were not significantly different from those without. Figure 5 compares the spectra between the photoreceptors with and without negative-going responses. The spectra in the receptors with negative-going responses are clearly narrower. (Here we use response amplitude instead of sensitivity because the negative-going parts are clearly seen in the response curves.)

**Figure 6:**
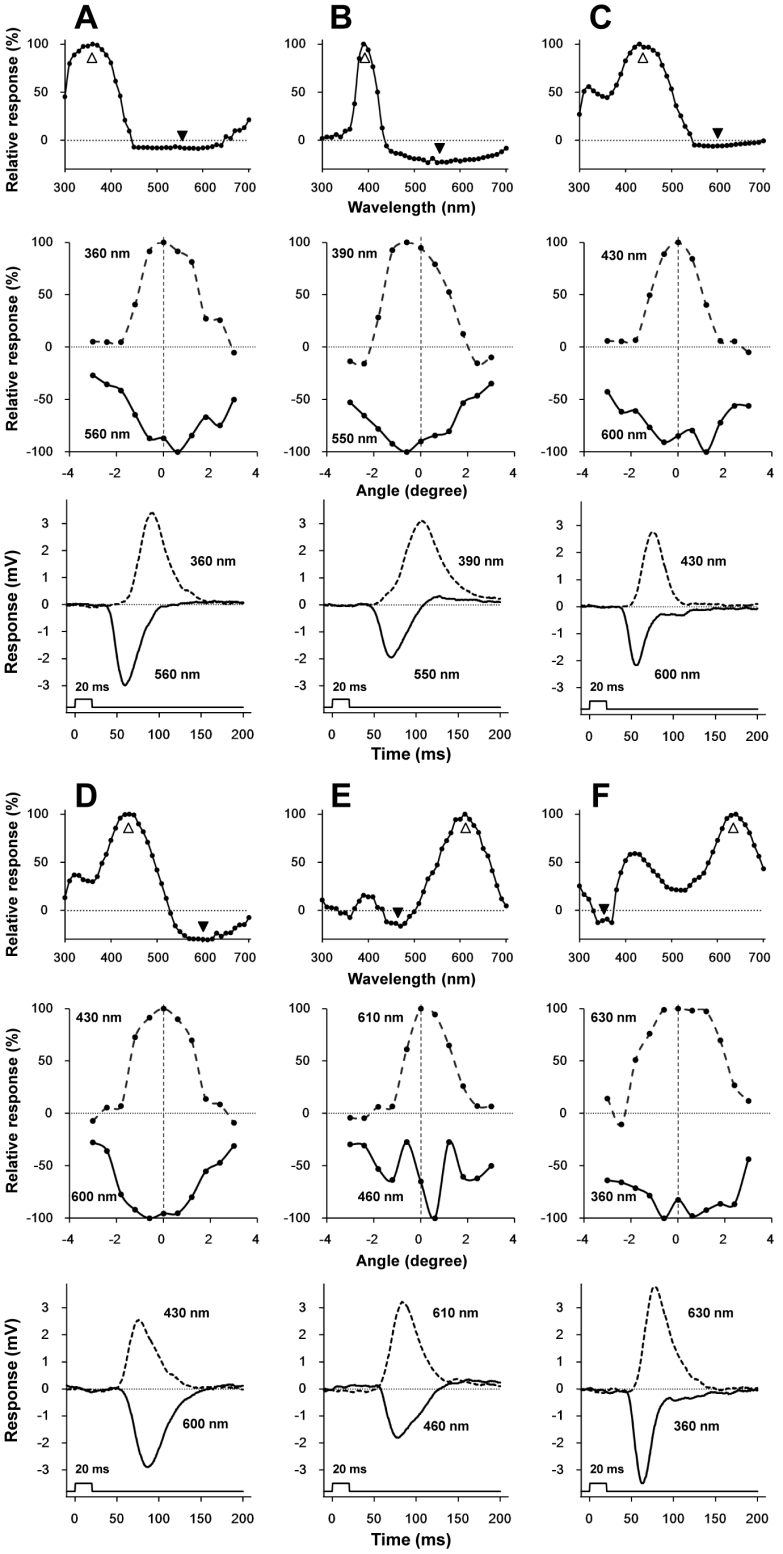
Analysis of negative-going responses. The *top*, *middle*, and *bottom rows* respectively show the spectral response, the angular response at the positive and negative peak wavelengths, and the positive and negative impulse responses. White and black arrowheads respectively indicate the wavelengths to record the positive and negative angular responses and the impulse responses. (A) UV receptor. (B) V receptor. (C) B receptor I. (D) B receptor II. (E) R receptor. (F) dR receptor. Except for the B receptor II (D), the τ_p, negative_ is faster than τ_p, positive_.

The angular responses as well as the time-to-peak of impulse responses (*τ*_p_) were measured at two wavelengths eliciting the largest positive and negative responses in 23 photoreceptors, six of which are shown in Figure 6. For example, Figure 6A shows the responses of an UV receptor with its spectral response (*top*), angular responses (*middle*) and impulse responses (*bottom*). The acceptance angles predicted from the angular responses (half-maximum of the angular response function) are not very different between the positive and negative peaks (*middle panels*). In the R receptor (Figure 6E), the peak of the angular response function at 460 nm (negative) is shifted about 0.6 deg from the peak at 610 nm (positive), and the profile is modulated. The *τ*_p_ values in the negative responses were smaller than those in the positive responses except for a subset of B receptors, which we termed as B receptor II (Figure 6D, see also Table 1).

**Table 1:** Temporal impulse responses of the photoreceptors of *Troides aeacus formosanus* to positive-peak and negative-peak wavelength stimulation.

Receptor type(number of data)	*τ*_p, positive_±SE (ms)	*τ*_p, negative_±SE (ms)	Δ*t*±SE (ms)
UV receptor (n = 5)	86.95±5.42	66.19±14.28	20.76±5.30
V receptor (n = 2)	91.33±11.07	69.13±1.94	22.20±9.20
B receptor I (n = 5)	76.05±3.81	64.20±9.71	11.85±3.29
B receptor II (n = 5)	70.85±3.89	75.79±6.55	−4.94±1.58
R receptor (n = 2)	81.65±1.34	72.73±3.36	8.93±1.43
dR receptor (n = 4)	78.85±7.14	64.78±9.69	14.08±1.94

*τ*_p_, time from the onset of the stimulus to the maximal response amplitude, Δ*t* = *τ*_p, positive_-*τ*_p, negative_. Values are means±SE.

For individual examples, see Figure 6.

## Discussion

### Diversification of spectral receptors by optical filtering

We identified nine spectral receptor types in *Troides aeacus formosanus*, which is the most among butterflies studied to date [8], [12], [16], [20]. In *Papilio xuthus*, five visual pigment opsins are expressed in the compound eye, which are R360 (PxUV), R460 (PxB), R515 (PxL2), R545 (PxL1), and R575 (PxL3) [8], [21]. Assuming a similar situation in *Troides*, we fitted the predicted absorption spectra of the visual pigments based on a template [22] to the spectral sensitivities of the *Troides* photoreceptors. The sensitivities of UV, B, BG, G (sG and dG) receptors matched reasonably well with the absorption spectra of R360, R440, R510, and R540 (Figure 2A–D), respectively. The spectral origin of these receptors is therefore attributed to the visual pigment absorption spectra.

However, the sensitivities of the V, O, R, and dR receptors are narrower than the predicted spectra of any visual pigments (Figure 2A, E, F). These sensitivities are most likely produced by the spectral filtering effect of the fluorescing and red pigments that characterize three ommatidial types (Figure 1).

The sensitivity profile of the *Troides* V receptors is similar to that of the V receptors in *Papilio* [23]. The *Papilio* V receptors express an R360 visual pigment, PxUV, and exist in the UV-fluorescing ommatidia. The fluorescing material, 3-OH retinol, absorbs 330 nm light and strongly suppresses the sensitivity of the R360-containing photoreceptors in the shorter wavelength region, shifting its peak sensitivity at 400 nm [23], [24]. Similar filtering is probably happening in the eyes of *Troides*, implying that the V receptors are localized in the fluorescing type II ommatidia (Figure 1). The absence of the secondary peak in the sG receptor can probably be attributed to the UV-absorbing function of the fluorescing pigment in type II ommatidia as in the case of *Papilio* [23]. The nature of the fluorescing pigment should be confirmed directly in the *Troides* eye.

Assuming that the eyes of *Troides* express a visual pigment similar to the *Papilio* PxL3 [25], an R575, we superimposed a predicted absorption spectra of R575 and R580 onto the spectral sensitivities of the O (Figure 2E), R and dR receptors (Figure 2F). However, none of them matched the predicted absorption spectra. We also superimposed predicted absorption spectra of R600 (Figure 2F), R610, R620 and R630 to the R and dR receptors, but none of them matched any spectral sensitivities either.

This indicates that the contribution of the red perirhabdomal pigments must be crucial. In *Pieris rapae*, the 620 nm-peaking R receptors and 640 nm-peaking dR receptors are all proximal receptors sharing an R560 visual pigment with the green-sensitive distal photoreceptors. What shifts the peak sensitivities in the R and dR receptors is the filtering effect of the red and deep-red perirhabdomal pigments [26], [27]. In *Papilio*, the narrow spectral sensitivity of the 600 nm-peaking R receptors is attributed to the optical interaction of an R575 and the red perirhabdomal pigments [25]. Judging from the eye fluorescence and the perirhabdomal pigmentation (Figure 1C, D) as well as the dye-injection experiments, the *Troides* R receptors are in the proximal tier of type I ommatidia that are weakly red-pigmented (Figure 3), while the dR receptors are in the proximal tier of type II ommatidia that are strongly red-pigmented (Figure 4). The O receptors are proximal receptors in the unpigmented type III ommatidia. This conclusion of course awaits the analyses of visual pigment opsins in *Troides*.

### The origin of negative-going responses

Some photoreceptors exhibit negative-going responses, which also sharpen the main response profiles (Figure 5). Similar responses of photoreceptors have been reported in the locust *Schistocerca gregaria* [10], the honey bee *Apis mellifera* [28], and in the butterfly *Papilio aegeus* [11], [12], although the origin of these responses is not necessarily clear. Shaw [10] proposed a model to explain the opposing currents in locust photoreceptors. The basic idea of his model is that the high external resistance of photoreceptors causes the extracellular return current to pass through the terminals of the neighboring receptors [10], [29], [30]. The direction of the return current is opposite to their own photocurrents. This results in hyperpolarization of the neighboring receptors and opposes their original response to light. Shaw's model was modified and applied to *Papilio aegeus* [12] to explain the photoreceptor spectral opponency, which confirms the passive lateral electrical inhibition among different receptors in the same ommatidium.

To address the origin of the negative responses in *Troides*, we measured the angular responses as well as the time-to-peak (*τ*_p_) of the impulse responses at two wavelengths, each eliciting a positive or negative response (Figure 6, see also Table 1). The normalized angular responses of the positive and negative responses indicated that the observed phenomenon was basically what was happening within a single ommatidium. One exception is the R receptor shown in Figure 6E. When stimulated at 460 nm, eliciting a negative response, the angular response function is modulated with a peak shift of about 0.6 deg. Assuming that the interommatidial angle of *Troides* is 0.5–1.0 deg [31], the modulation of the angular response function may be due to interommatidial interactions between photoreceptor terminals across the cartridges [32].

In most cases the time-to-peak of the negative responses, *τ*_p, negative_, were faster than those of the positive responses, *τ*_p, positive_, (Figure 6A, B, C, E, F, Table 1). These faster negative responses cannot simply be explained by returning photocurrents as described in Shaw's model [10]. Matić [12] also recorded faster negative responses in *Papilio aegeus*, and attributed them to neighboring receptors with different spectral sensitivities receiving stronger stimulation, resulting in a fast and strong ERG-like negative deflection. This could also be the case in *Troides*.

Another possible mechanism for the fast negative responses may be synaptic interactions. The photoreceptors feed information to the second order visual interneurons in the lamina (large monopolar cells, LMCs) via histaminergic synapses[33]. The LMCs express histamine-activated chloride channels, which produce hyperpolarization in the LMCs in response to photoreceptor depolarization [33], [34]. The LMC responses are faster than the photoreceptor potentials [35], which is probably due to the high gain of the synapses. We have found in *Papilio xuthus* that the photoreceptors originating from a single ommatidium bundled in a lamina cartridge are mutually connected by synapse-like structures [36]. The connections are found between photoreceptors of identical spectral sensitivities, and also between photoreceptors of different spectral sensitivities. Supposing that these structures also exist also in *Troides*, and also that the structures between different spectral receptors are histaminergic synapses similar to the photoreceptor-LMC synapses, then a photoreceptor stimulated at its peak wavelength of the spectral sensitivity would elicit fast hyperpolarization in the post-synaptic photoreceptors in the same cartridge. In the present study we penetrated the photoreceptors in the retina, i.e., in the cell body region. However, the negative responses should be more clearly seen in the lamina if the responses are in fact due to the possible synaptic interactions between photoreceptor terminals.

Although not found in butterflies, the photoreceptors might be connected via gap junctions like in flies [37], [38]. If the gap junctions have rectifying properties [39], then they could also contribute to producing negative responses in neighboring photoreceptors under certain circumstances.

### Functional implications

We identified at least nine spectrally distinct photoreceptors in the eye of *Troides aeacus formosanus*, which is not only the most among butterflies studied so far, but also the first for butterfly species in the tribe Troidini (Papilionidae). *Troides* butterflies are frequent flower visitors. A rich variety of spectral receptors indicates that they strongly rely on color discrimination.

The possible electrical interaction between photoreceptors raises the question of whether and how color information processing is improved by the mechanism in the visual system at the initial stage. Color opponency is one of the most important neural mechanisms for processing color information [40]. Several insect species have also been demonstrated to have color opponent mechanisms in their visual system [41]–[46], but they are all found in higher-order neurons in the medulla and/or lobula.

The negative-going responses of the *Troides* photoreceptors clearly sharpen their spectral sensitivities. If the fast negative responses originate from the inhibitory synaptic interactions between photoreceptors in the lamina, then this mechanism could be a pre-processor for the second order LMCs. LMCs would thus receive input from the photoreceptors whose spectral sensitivities are even narrower than the ones recorded in the retina. Sharpening the LMC spectral sensitivity has also been demonstrated in a dragonfly, which may be due to similar mechanism [47]. The narrower spectral sensitivity as well as the diverse receptor types may enhance the ability of color discrimination.
